# Correction: Multi-stage feature selection (MSFS) algorithm for UWB-based early breast cancer size prediction

**DOI:** 10.1371/journal.pone.0251679

**Published:** 2021-05-06

**Authors:** V. Vijayasarveswari, A. M. Andrew, M. Jusoh, R. B. Ahmad, T. Sabapathy, R. A. A. Raof, M. N. M. Yasin, S. Khatun, H. A. Rahim

The authors are listed out of order. Please view the correct author order, affiliations, and citation here:

V. Vijayasarveswari^1^, A.M. Andrew^1^, M. Jusoh^1^, R.B. Ahmad^1^, T. Sabapathy^1^, R.A.A. Raof^1^, M.N.M. Yasin^1^, S. Khatun^2^, H.A. Rahim^1^

**1** Advanced Communication Engineering (ACE) Centre of Excellence, Universiti Malaysia Perlis, Kangar, Perlis, West Malaysia, **2** Faculty of Electrical & Electronic Engineering, Universiti Malaysia Pahang, Pekan, Pahang

Vijayasarveswari V, Andrew AM, Jusoh M, Ahmad RB, Sabapathy T, Raof RAA, et al. (2020) Multi-stage feature selection (MSFS) algorithm for UWB-based early breast cancer size prediction. PLoS ONE 15(8): e0229367. https://doi.org/10.1371/journal.pone.0229367

There are errors in the Funding statement. The correct Funding statement is as follows: The study was supported by Fundamental Research Grant Scheme (FRGS), Ministry of Education Malaysia under grant number: FRGS/1/2019/TK04/UNIMAP/02/3. No additional external funding was received for this study.
