# Supplementary material for: Multi-stage feature selection (MSFS) algorithm for UWB-based early breast cancer size prediction
Source: PLoS One. 2020 Aug 13;15(8):e0229367. doi: 10.1371/journal.pone.0229367 (PMC7425918; doi:10.1371/journal.pone.0229367)
Supplement: Attachment — Submitted filename: Response to Reviewers.pdf [file pone.0229367.s001.pdf]

Plos One Journal Revision

No:	Review Comments:	Revision Made/ Justification:	Remarks:
1	**<u>Editor</u>**<br><br>A rebuttal letter that responds to each point raised by the academic editor and reviewer(s). This letter should be uploaded as separate file and labeled 'Response to Reviewers'.	The rebuttal letter that responds to each point raised by the academic editor and reviewer(s) are included. The letter is uploaded as separate file and labeled ‘Response to Reviewers’.	
2	**<u>Editor</u>**<br><br>A marked-up copy of your manuscript that highlights changes made to the original version. This file should be uploaded as separate file and labeled 'Revised Manuscript with Track Changes'.	The changes made to the original version is highlighted in orange colour in the revised manuscript. Since we used Overleaf as LATEX editor, track change feature is not available. Thus, we used orange highlight to indicate the revision made. The file labeled 'Revised Manuscript with Track Changes'.	
3	**<u>Editor</u>**<br><br>An unmarked version of your revised paper without tracked changes. This file should be uploaded as separate file and labeled 'Manuscript'.	The unmarked version of the revised paper without tracked changes is labeled as 'Manuscript'.	
4	**<u>Editor</u>**<br><br>Please ensure that your manuscript meets PLOS ONE's style requirements, including those for file naming.	The manuscript is formatted using Plos One LATEX style formatting.	If the manuscript is formatted using old version of Plos One LATEX formatting, the authors will reformat again with the new Plos One LATEX formatting in upcoming revision.
5	**<u>Editor</u>**<br><br>Please upload a copy of Figure 9 to which you refer in your text on page xx. If the figure is no longer to be included as part of the submission please remove all reference to it within the text.	In the previous version, Figure 8 (part 2) supposed to be Figure 9. It was mistakenly wrote as Figure 8 instead of Figure 9. Figure 9 is changed and included in this revision.	
6	**<u>Editor</u>**<br><br>Please include your tables as part of your main manuscript and remove the individual files. Please note that supplementary tables (should remain/ be uploaded) as separate "supporting information" files.	The tables are included as a part of the main manuscript. Thus, the tables are removed from the individual files.	

7	<u>**Editor**</u><br><br>Please update your submission to use the PLOS LaTeX template.	The manuscript is formatted using Plos One LATEX style formatting.	If the manuscript is formatted using old version of Plos One LATEX formatting, the authors will reformat again with the new Plos One LATEX formatting in upcoming revision.
8	<u>**Editor**</u><br><br>Please upload your figure files to the Preflight Analysis and Conversion Engine (PACE) digital diagnostic tool.	The figures are converted using PACE tool	
9	<u>**Reviewer 1**</u><br><br>Usually sensitivity/specificity are defined following these assumptions:<br>negative cases: breast with NO lesions<br>positive: breast with lesions<br><br>Do you consider also breast with NO lesions in your experiments?<br><br>how many data of breast with NO lesions are you considering?<br><br>If you are not considering breast with NO lesions, how can you calculate specificity?<br><br>A deeper description/ investigation/discussion of sensitivity, specificity and accuracy is required, especially using a medical point of view.	6 possible outputs of the classifier are 5 sizes of tumor + non-existence. The authors missed out in the paper the information of total non- existence samples added for final classification (750 samples). But, no changes in the classification accuracy of the classifiers presented in the paper as the presented table considers 7500 samples (6750 size samples + 750 non- existence samples). The changes are updated in the revision. 7500 data samples, and 750 data samples for each fold.<br><br>TP: correct classification of cancer size<br>TN: correct classification of non-existence<br>FN: incorrect classification of non-existence<br>FP: incorrect classification of cancer size	The discussion on sensitivity, specificity and accuracy is added in the manuscript.<br><br>**(Lines 222, 226, 234 – 244)**
10	<u>**Reviewer 2**</u><br><br>However, I felt that while reading the manuscript their is lack of further explanation, especially the part where the cancer detection method is not explained well.	The cancer detection method is explained in the paper.	A new figure (**Figure 13)** is added to explain about the cancer detection method implemented in this research.<br><br>**(Lines 287 – 292, 296, 304)**

11	<u>**Reviewer 2**</u><br><br>The statistical procedure is explained, however is not supported by some real data. A further explanation with support from some real data, will certainly increase the overall impact of the manuscript.	The work presented in this paper is a preliminary work on statistical procedure involved in the framework. Currently, our research is dealing with real data on patients. Thus, the work on real data will be covered in our future papers.	
12	<u>**Reviewer 3**</u><br><br>What do you mean by "researchers used either real-time machines (...) or machine learning to analyze UWB signals" while citing ref [1]?	Based on [1], usually, the UWB signals can be analyzed using machines or algorithm. The common machine used to analyze UWB is Vector Network Analyzer, which contains both a source, used to generate a known stimulus signal, and a set of receivers, used to determine changes to this stimulus caused by the device-under-test (DUT) such as UWB based system.	
13	<u>**Reviewer 3**</u><br><br>In the phantom section. Glass is giving a proper shape to the phantom, but is material also mimicking the skin dielectric properties? Maybe call it skin is not exact.	Yes, it is statistically validated that the material dielectric properties are same as the skin.	The properties of the breast phantom are added (**Table 1**).<br><br>**Lines 22, 40 – 42**
14	<u>**Reviewer 3**</u><br><br>Any details on used antenna?	Yes, it is added in the paper.	The details of UWB antenna used in this research is added (**Table 2**).<br><br>**Lines 48 – 49**
15	<u>**Reviewer 3**</u><br><br>Are the antennas touching the phantom?	The antennas are not touching the phantom.	The setup of the antenna transmitter and receiver is added (**Figure 4**).<br><br>**Line 36, 42**
16	<u>**Reviewer 3**</u><br><br>Antennas are placed in only one position. Have the authors thought on a multi-view approach?	For the work in this paper, antennas are placed in only one position. Currently, we are improving the signal and efficiency of the detection using multi- view approach.	

17	**<u>Reviewer 3</u>**<br><br>Did the authors compare their proposed method to a known one using the same dataset? (Maybe is already answered in Table 6). My question is if same dataset is used on both cases.	No, since this work is the improvement of the approach discussed in [1] and [31] (Table 6). Work [31] uses only 240 data samples, which are much lesser than the samples used in this paper. Our current work is focusing on improving our current system with multi- view approach proposed in work [1] and [31].	
18	**<u>Reviewer 3</u>**<br><br>Classifier is giving information on size only. Which is the idea in giving a 3D image of this if position is not known? Is it to give an idea on relative size to the breast?	The presented GUI in 2D and 3D environment are combination of few research arms on early breast cancer detection, consist of cancer existence, cancer size (covered in this paper), location detection, and cancer type detection.	The discussion is added in the paper.<br><br>**Figure 13**<br><br>**(Lines 287 – 292, 296, 299, 304)**
19	**<u>Reviewer 3</u>**<br><br>Did the authors try with a case that is out of the ones already defined? I mean: What happens if a testing data with a tumour of a different size is used? (let's say 2.5 mm or 1 mm)	The aim of the research is to detect the cancer tumor at early stage which is usually 2 mm to 6 mm. This case is covered in another arm of the research on detecting the cancer existence. Having proportion of size (i.e. 2.5 mm) actually can still be detected by the system. It will be either detected as 2 mm or 3 mm, based on the data filtered statistically by the algorithm. Having TN and FN values in the accuracy, specificity and sensitivity calculation can help in minimizing the errors and misclassifications.	
