# Supplementary material for: Multi-stage feature selection (MSFS) algorithm for UWB-based early breast cancer size prediction
Source: PLoS One. 2020 Aug 13;15(8):e0229367. doi: 10.1371/journal.pone.0229367 (PMC7425918; doi:10.1371/journal.pone.0229367)
Supplement: Attachment — Submitted filename: Response to Reviewers.pdf [file pone.0229367.s002.pdf]

Plos One Journal Revision

No:	Review Comments:	Revision Made/ Justification:	Remarks:
1	**Editor**<br><br>The manuscript has been improved. However, there are still some major points that should be addressed. Please work on them and revise the manuscript accordingly.	The manuscript has been revised. The major points are addressed to give a better understanding to the readers.	
2.	**Reviewer 1**<br><br>Please update the ref, including for example:<br><br>Aldhaeebi, M.A.; Alzoubi, K.; Almoneef, T.S.; Bamatraf, S.M.; Attia, H.; Ramahi, O.M. Review of Microwaves Techniques for Breast Cancer Detection. Sensors 2020, 20, 2390.<br><br>Rana, S.P., Dey, M., Tiberi, G. et al. Machine Learning Approaches for Automated Lesion Detection in Microwave Breast Imaging Clinical Data. Sci Rep 9, 10510 (2019). https://doi.org/10.1038/s41598-019-46974-3	The references are updated in the manuscript.	**References [42], [43]**
3.	**Reviewer 3**<br><br>Authors declare in the response: "it is statistically validated that the material dielectric properties are same as the skin.". But Glass has, as declared in table 1, an epsilon_r between 3.5 and 10, while sigma value is considered negligible. These are normal values for glass. When we check skin values in [10], at the central frequency of 4.3 GHz epsilon_r is greater than 30 and sigma is almost 3 S/m. These values are confirmed in http://niremf.ifac.cnr.it/tissprop/ (eps= 36.342 and sigma=2.5443 S/m for dry skin).	We are sorry because our previous explanation for this question was incomplete. We agree with the reviewer that the dielectric properties are not same for glass and skin. However, experimental set up from previous works from M.T.Islam et al (Scientific Report, IF: 3.998) [44] and Salehin Kibria et al (IEEE Access, IF: 3.745) [45] has shown that certain type of containers are still being used to hold the breast phantom during the measurement. Furthermore, the results are normalized after the measurement are carried out. Therefore, the containers permittivity either glass or any other materials will have different level of signal strength, but after normalization, it should be the same. Thus, our proposed framework still can work perfectly	**References [44], [45] are added.**<br><br>**The paper is revised with information on this in Line 42-50.**

		with the effect with or without glass. Changes are made in the revised manuscript to clarify this. Additional references are added to give a better understanding for the readers on the effect of normalization techniques to rectify the issue. To avoid confusion, the skin and glass are removed from Table 1.	
4.	**Reviewer 3**<br><br>Antenna Details. In Table 2 seems that Patch Description and Substrate Description contain the same information.<br>I expected a scheme, photo or drawing of the antenna, or a S11 plot in the frequency range of interest.	The detailed information of the scheme, photos, drawing of the antenna, scattered UWB waveform, Reflection co-efficient (S11) comparison of the proposed and commercial antennas, as well as the other related details have been published previously by the authors in Journal of Medical Imaging and Health Informatics (IF 0.659) (Reference 18). Based on the suggestion from Reviewer 3, the information is added.	**Additional information is added in Line 57-58.**
5.	**Reviewer 3**<br><br>My question related to the comparison with a different algorithm was oriented to give a better glance at how your method works compared to existing and validated methods.	Based on the advice from Reviewer 3, the comparison with other existing validated methods were added. In order to do that, the previous Table 8 is removed and replaced with more detailed Table 8 and Table 9. Table 8 shows the results of data from previous researches fed into the proposed method in this paper. Table 9 shows the results of the data from this research fed into the existing validated methods.	**Clarification on this is added in Line 287-294.**
